# Concordant association validates MGMT methylation and protein expression as favorable prognostic factors in glioma patients on alkylating chemotherapy (Temozolomide)

**DOI:** 10.1038/s41598-018-25169-2

**Published:** 2018-04-30

**Authors:** Arshad A. Pandith, Iqbal Qasim, Wani Zahoor, Parveen Shah, Abdul R. Bhat, Dheera Sanadhya, Zafar A. Shah, Niyaz A. Naikoo

**Affiliations:** 1 Advanced Centre for Human Genetics, Sher-I-Kashmir Institute of Medical Sciences (SKIMS), Srinagar, J & K India; 2 Department of Pathology, SKIMS, Srinagar, J & K India; 3 Department of Neurosurgery, SKIMS, Srinagar, J & K India; 4 School of Life and Basic Sciences, Jaipur National University, Jaipur, 302025 India; 5 Immunology and Molecular Medicine, SKIMS, Srinagar, J & K India; 6 Department of Biotechnology, Higher Education Department, Cluster University, Srinagar, J & K India

## Abstract

O^6^-methylguanine-DNA methyltransferase (MGMT) promoter methylation and its subsequent loss of protein expression has been identified to have a variable impact on clinical outcome of glioma patients indicated for chemotherapy with alkylating agents (Temozolomide). This study investigated methylation status of MGMT gene along with *in situ* protein expression in malignant glioma patients of different histological types to evaluate the associated clinical outcome vis-a-vis use of alkylating drugs and radiotherapy. Sixty three cases of glioma were evaluated for MGMT promoter methylation by methylation-specific PCR (MS-PCR) and protein expression by immunostaining (IHC). Methylation status of MGMT and loss of protein expression showed a very high concordant association with better survival and progression free survival (PFS) (p < 0.0001). Multivariate Cox regression analysis showed both MGMT methylation and loss of protein as significant independent prognostic factors in glioma patients with respect to lower Hazard Ratio (HR) for better OS and PFS) [p < 0.05]. Interestingly concordant MGMT methylation and lack of protein showed better response in TMZ therapy treated patient subgroups with HR of 2.02 and 0.76 (p < 0.05). We found the merits of prognostication of MGMT parameters, methylation as well as loss of its protein as predictive factors for favorable outcome in terms of better survival for TMZ therapy.

## Introduction

Malignant gliomas are among the most prominent brain tumors comprising of many types in particular Astrocytoma, Oligodendroglioma and Glioblastoma multiforme (GBM) which represent most frequent histological class with aggressive mode and propensity to invade adjacent tissue with a clinical relevance to treatment sensitivity^1,2^. Researchers have unraveled a good understanding of the biological science of these tumor types and open to a good extent glioma tumorigenesis, proliferation, and invasion^3^. Malignant glioma could develop in the higher ages, the high incidence being mosly within the 5^th^ and 6^th^ decades of life. The average incidence of malignant glioma is about 5 per one lac per year. As per a detailed study by Dhar *et al*. (2010: data unpublished) in valley of Kashmir (North India), male: female ratio is reported 1.47:1 which occur most commonly among age group between 41–50 years. Among common brain tumors, the frequency of malignant glioma accounts for 51.3% of all the cases wherein GBM is the commonest affecting 49.5% of cases in our region.

In malignant gliomas apart from chromosomal aberrations like chromosome 1 P/19q deletion, genetic and epigenetic derangements have been found^4^ but the clinical outcome of the most glioma-associated molecular alterations still remain unclear^5^.

Although there has been considerable advances in the field of brain tumorigenesis, the prognosis of patients with glioma still remains very poor^6^ wherein particularly, the survival time of the patients with GBM ranges from 12 to 15 months^7^.

Treatment of malignant glioma encounters resistance to different chemotherapeutic agents in particular with Temozolomide (TMZ), one of the alkylating agents which are highly reactive molecules to render cell death by binding to DNA^8,9^. Alkylating agents are encumbered to cross-link double-stranded DNA by ubiquitously expressed DNA-repair enzyme O^6^-*methylguanine-DNA methyltransferase* (MGMT) encoded by MGMT gene located at chromosome 10q26^10^. Owing to the stability of *MGMT* gene, it is not commonly mutated or deleted, but a lack of MGMT may be caused by the epigenetic kick as methylation do not alter the genetic information of the cell. Methylation of DNA represents such epigenetic modifications in humans^11^ and it plays a vital role in tumorigenesis. Methylation of the CpG Island halts transcription of MGMT gene causing the altered expression of the product which compromises its efficiency to repair alkylation of O^6^-methylguanine^12–15^. This has been substantiated with demethylating drugs which reverses the expression of the *MGMT* gene in same cells *in vitro* treatment^13,14^.

The MGMT, a potent DNA repair enzyme repairs alkylating lesions of the DNA by knocking out mutagenic adducts from the O^6^ position of guanine as usually caused by the Temozolomide, a chemotherapeutic agent used to treat Glioma^13^. This influences the treatment in cells with intact MGMT gene and confers drug resistance. On the other hand the therapeutic response to alkylating agents is improved in tumor cells with hypermethylation in MGMT causing its silence resulting either in low levels or complete loss of MGMT expression^16^. This phenomena causes MGMT promoter methylation to correlate with a survival benefit in glioma patients treated with alkylating chemotherapeutics^17^. MGMT promoter methylation thus actually represents part of a genetic signature of chemotherapeutically sensitive gliomas^18–20^.

The MGMT levels show wide variations across different tumor types or among tumors of the same type. Methylation of the MGMT promoter is found in 35%–45% of malignant gliomas (WHO grades III and IV) and in about 80% of WHO grade II gliomas^17,21^. In this group of patients, deficiency of the enzyme may enhance the sensitivity of neuro-tumors to alkylating agents^22–24^ and the expected clinical outcome of the patients can be marginally enhanced. Because of this reason determination of the methylation pattern of the MGMT gene becomes mandatory in patients with malignant glioma.

Thus, evaluation of methylation status of the *MGMT* gene promoter by methylation specific polymerase chain reaction (MS-PCR) and the expression level of MGMT protein by Immunohistochemistry (IHC) in malignant glioma has come to be realized as one of the most requested molecular assays in clinical neuro-oncology. The present study was aimed to investigate the prognostic scope of MGMT by evaluating both its promoter methylation status and protein expression in a series of primary malignant glioma patients with varied histologies treated with alkylating agent, Temozolomide and radiotherpay.

## Results

This study included 63 confirmed cases of malignant glioma patients which were analyzed for MGMT gene methylation and its protein expression to observe the impact of alkylating chemotherapeutic agent Temozolomide on the OS and PFS of the patients. Among 63 glioma cases, 47(74.6%) were males as compared to 16 (25.5%) females. On stratifying malignant glioma cases, there were 32(53.9%) cases of Glioblastoma, 14(22.2%) Astrocytoma, 14(22.2%) Oligidendrioglioma and other types of glioma included 3(4.7%) cases. 45 (71.4%) and 42(66.7%) cases received both treatments successively and 58.8% received chemotherapy & radiotherapy concurrently. On follow up (around 36 months), 36 (57.1%) patients had died while as 27 (42.9%) were still alive up to last follow up. The clinico-pathological characteristics of the patients are detailed in Supplementary Table 1.

### MGMT promoter methylation status as analyzed by MS-PCR

Of the 63 glioma samples analyzed for pattern of methylation, 38 (60.3%) cases showed methylation in MGMT gene and 22 (39.7%) were unmethylated. In GBM alone, 17 (53.1%) cases were with methylated sequences in comparison to 15 unmethylated (46.9%) followed by Astrocytoma 09 (64.2%) and Oligidendrioglioma with 12 (85.7%) methylated DNA sequences. A proportion of samples (6 of 63: 9.5%) analyzed for methylation were observed to contain a mixed profile with both methylated and unmethylated sequences. The pattern of methylation status among the different age groups and gender were observed to be insignificant (p > 0.05). The status of methylation did not associate with any clinico-pathological characteristics as shown in Supplementary Table 1.

### MGMT protein expression as analyzed by IHC

**Figure 1:**
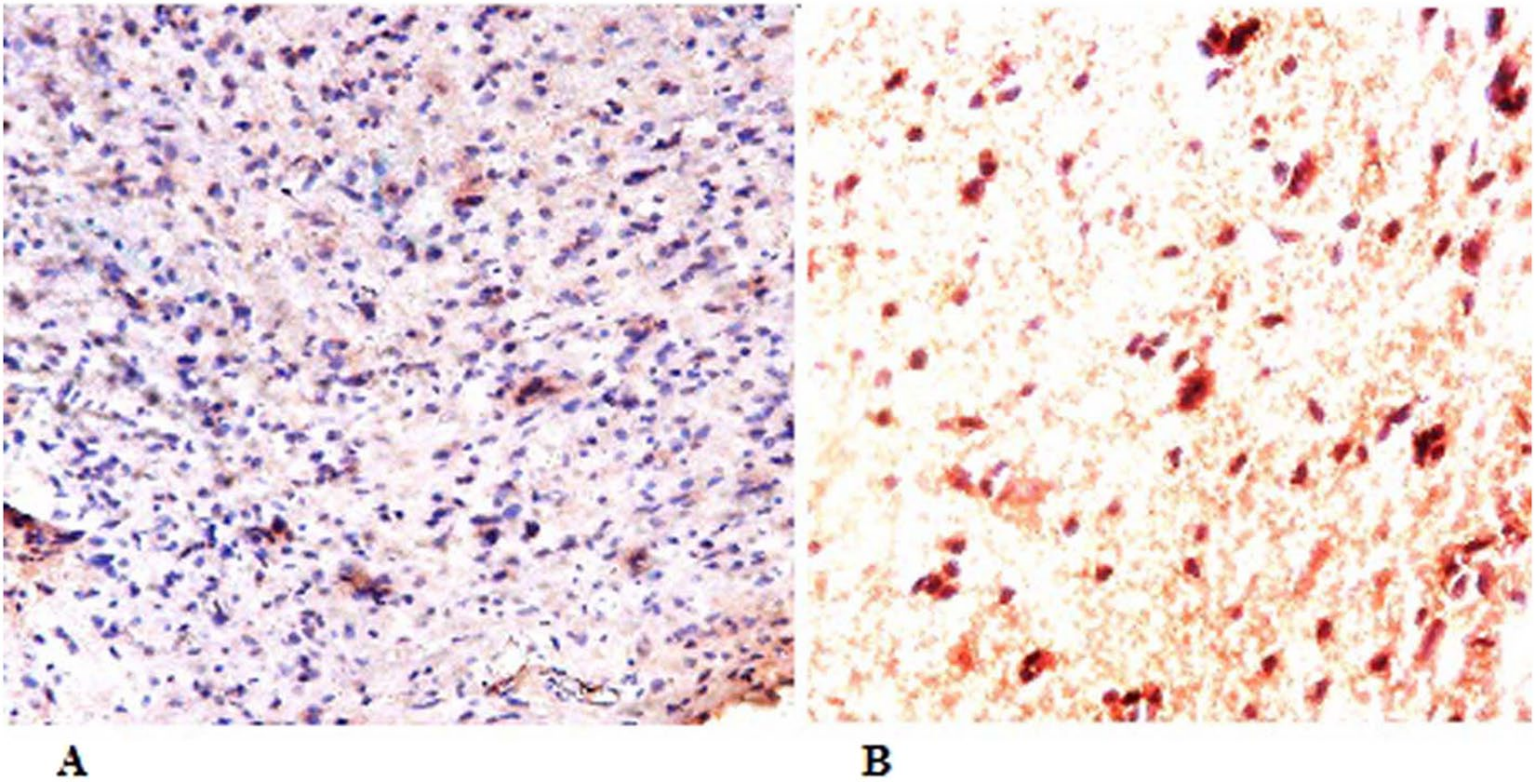
Protein expression of MGMT by Immunohistochemistry (IHC). (**A**): MGMT showing complete expression of the protein in tumor cell with unmethylated DNA. (**B**): Lack of MGMT protein expression with its Methylated MGMT promoter in the majority of tumor cells.

Protein expression of MGMT by IHC was done for all the patients whose methylation status was known. Loss of protein expression was found in 36 cases (60%) while as the 25 (40%) patients had intact MGMT with complete expression Fig. 1A,B. The quantum of expression in malignant gliomas varied to a great extent wherein tumors with intact MGMT showed higher levels of expression than adjacent normal tissue. No significant difference in protein expression levels were detected in different age groups, gender or among any histological types of glioma (p < 0.05). The detailed information of protein expression status of MGMT is given in supplementary Table 2.

### Correlation of MGMT Methylation and its Protein Expression

MGMT methylation and its protein expression were performed on same series of glioma samples. Of the 24 samples with intact MGMT protein expression, 17 (70.8%) were unmethylated and 7 methylated (29.2%) while as samples with loss of protein expression, 31 (86.1%) were methylated in comparison to 5 (13.9%) unmethylated. The results showed very high concordance and significant association between the pattern of methylation and protein expression (p < 0.0001). Among various histological types of glioma, GBM showed concordant pattern between methylation and protein expression with significant association (p < 0.001) (Table 1).

**Table 1:** Association of MGMT methylation and loss of MGMT protein expression in different parameters of Glioma patients.

Characteristics	No. of samples	Methylated MGMT	Unmethylated MGMT	P value
**Overall tumors**				
Expression	25	07	17	<0.0001
No expression	36	31	05	
**Tumor Type**				
***Glioblastoma***	32			
Expression		03	11	0.000
No expression		16	02	
***Astrocytoma***	14			
Expression		01	03	0.2
No expression		07	03	
***Oligidendrioglioma***	14			
Expression		03	01	0.9
No expression		08	02	
**Grade** ***/II (Low)***	08			
Expression		01	01	0.9
No expression		05	01	
***Grade III***	24			
Expression		04	04	0.1
No expression		13	03	
***Grade IV (High)***	28			
Expression		01	11	0.00
No expression		14	02	
**Dead**	31			
Expression		03	14	0.00
No expression		13	01	
**Alive**	27			
expression		03	04	0.03
No expression		17	03	

When stratified in various grades, MGMT protein expression level for high grade was significantly higher in samples with unmethylated sequences, whereas loss of expression was associated in high accordance with methylated group (p < 0.001). Association of other characteristic features of glioma patients and their correlation with MGMT methylation and protein expression is given in Table 1.

### Correlation of MGMT methylation and protein expression with Temozolomide status

A significant correlation was noticed in patients on Temozolomide when results were stratified in terms of methylation status and protein expression (p < 0.05). Also Temozolomide correlated with GBM and high grade tumors with significant concordance in MGMT methylation and protein expression (p < 0.05). Correlation of MGMT methylation and protein expression with Temozolomide response status for other characteristics of glioma patients is given in Supplementary Table 3.

### Impact of MGMT promoter methylation status and its protein expression on overall survival (OS) and progression free survival (PFS)

At a mean follow-up time of 36 months for 63 patients, 36 (57.1%) were known to be dead and 27(42.9) were alive. Kaplan-Meier (KM) survival analysis was performed to evaluate any possible association between various clinico-pathological characteristics, with respect to overall survival (OS) and progression free survival (PFS) of patients. A marked difference in both OS and PFS were observed in histological types of malignant glioma wherein GBM accounted for less OS and PFS of 13.8 (95%; 10.7–16.9) and 10.0 (95%; 7.1–12.8) months compared to other groups with significantly higher OS and PFS [OS Astrocytoma 19.5 (95% 12.7–26.3), OS Oligidendrioglioma; 62.59 (50.7–74.4), p = 0.001; PFS Astrocytoma 16.2 (9.1–23.3), Oligidendrioglioma 32.2 (27.4–37.1); P = 0.001] as shown in Table 3. Similarly patients with different grades showed significant differences in OS and PFS (p < 0.05) (Table 2).

**Table 2:** Relation of various variables of Glioma patients with respect to survival and progression free survival.

Parameter	Mean OS	95% CI	P value	Mean PFS	95% CI	P value
**Tumor Type**						
Glioblastoma	13.813	10.7–16.9	0.001	10.0	7.1–12.8	0.001
Astrocytoma	19.595	12.7–26.3		16.2	9.1–23.3	
Oligidendrioglioma	62.592	50.7–74.4		32.2	27.4–37.1	
**Grade**						
I/II (Low)	27.533	20.5–34.4	0.01	23	15.8–30.1	0.016
III	40.511	27.5–53.4		22.475	16.7–28.1	
IV (High)	14.162	10.7–17.5		10.809	7.3–14.2	
**Chemotherapy**						
TMZ Given	41.119	30.8–51.3	0.000	24.623	20.8–28.3	0.000
TMZ Not Given	5.444	13.4–7.4		2.278	0.31–4.2	
**Radiotherapy**						
Given	40.27	30.1–50.4	0.000	24.049	20.2–27.8	0.000
Not Given	6.056	3.7–8.3		2.833	0.46–5.2	
**Chemo + Radio**						
Given	43.379	32.5–54.1	0.000	25.805	21.9–29.6	0.000
Not given	6.964	4.7–9.1		3.818	1.5–6.0	
MGMT Methylated	40.097	29.8–50.3	0.000	23.9	20.0–27.7	0.000
MGMT Unmethylated	6.75	3.8–9.62		3.2	0.59–5.80	
MGMT expression	18.226	7.0–29.4	0.000	8.952	3.0–3.04	0.001
MGMT No expression	36.255	25.4–47.0		22.602	2.0–18.5	
**Age**						
<50	38.816	26.4–51.2	0.025	21.8	16.3–27.2	0.022
≥50	16.432	12.1–206		14.007	9.3–18.6	
**Sex**						
Male	34.772	24.3–45.1	0.111	19.448	14.7–24.1	0.132
Female	15.715	11.8–19.6		12.883	9.0–16.7	

TMZ: Temozolomide.

**Figure 2:**
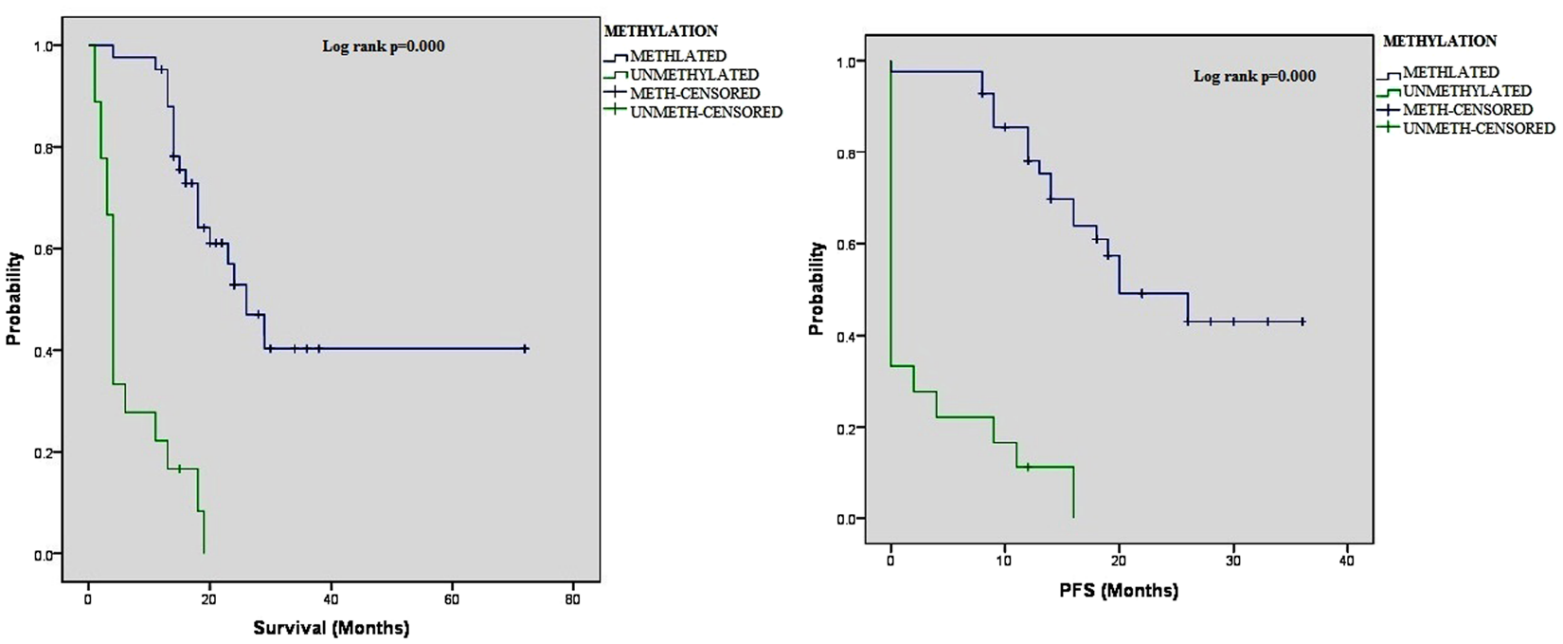
Association of MGMT promoter methylation with overall survival (OS) and Progression Free survival (PFS) of glioma patients as shown by KM analysis.

**Figure 3:**
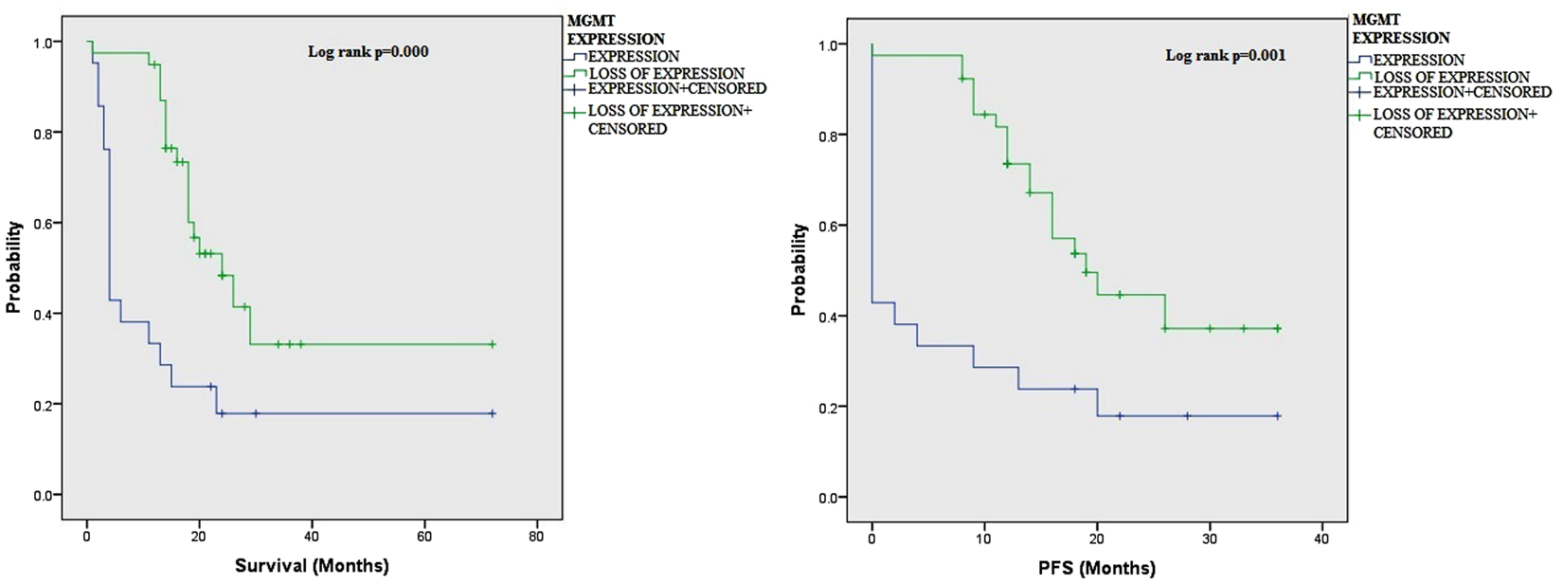
Association of MGMT protein expression with overall survival (OS) and Progression Free survival (PFS) of glioma patients as shown by KM analysis.

By Kaplan Meir (KM) analysis, OS and PFS of glioma patients were compared to both MGMT promoter methylation status and its protein expression by multivariate analyses (Table 2). MGMT methylation status showed a significantly higher OS and PFS of 40.0 months (95% C.I;29.8–50.3) and 23.9 months (95% C.I; 20.0–27.7) as compared to unmethylated group of patients with 6.7 months (95% C.I; 3.8–9.62) and 3.2 months (95% C.I; 0.59–5.8) respectively (log rank p = 0.000) as depicted in Fig. 2. Same significant concordance was observed in patient group on Temozolomide with loss of MGMT protein expression where OS and PFS were also seen markedly higher 36.25 months (95% C.I; 25.4–47.0) and 22.60 months (95% C.I; 2.0–18.5) as against those with intact MGMT protein having lower OS and PFS of 18.23 months (95% C.I; 7.0–29.4) and 8.96 months (95% C.I; 3.0–3.04) respectively with log rank p = 0.00 (Fig. 1 supplementary). Temozolomide (TMZ) therapy also showed significantly better survival response in glioma patients as compared untreated ones (p = 0.000) as shown in Fig. 3.

**Figure 4:**
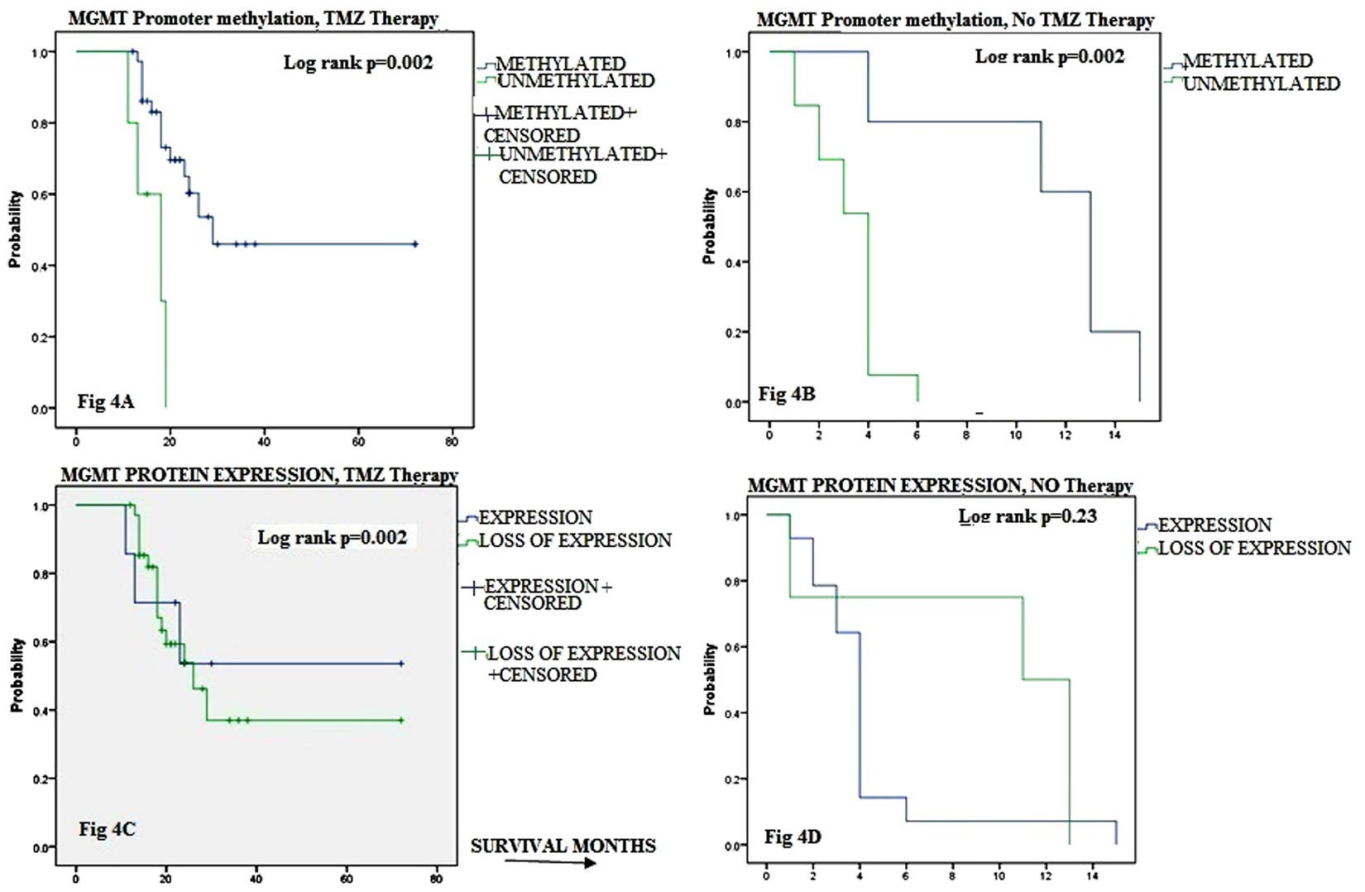
Comparison of MGMT promoter methylation and protein expression with overall survival (OS) of patients. KM analyses of OS for MGMT promoter methylation (**A**,**B**) or MGMT protein expression (**C**,**D**) of Glioma treated (**A**,**C**) and untreated (**B**,**D**) with TMZ therapy.

Owing to strong significant interactive concordance between MGMT parameters (methylation and protein expression) and TMZ therapy (p < 0.05), we deduced their association with OS in patients from subgroups treated and untreated with TMZ therapy (Fig. 4A–D). In the patient group treated with TMZ therapy, MGMT methylation strongly associated with longer OS of 44.12 months (95% C.I; 44.12–15.90; P = 0.002) as compared shorter OS of 15.9 months only in patients without TMZ therapy (95% C.I; 12.579–19.221). Similarly, patient cohort with lack of protein expression showed significantly longer OS of 37.35 months (95% C.I; 27.77–50.99; p = 0.002) when treated with TMZ therapy in contrast with shorter OS of 9.5 months only in untreated patients (95% C.8I; 3.87–15.13; P = 0.253). The difference among two groups of patients with differential MGMT protein expression who were treated with TMZ therapy and those who were not showed a significant difference (p < 0.05). Interestingly both MGMT parameters depicted significant concordance for better OS of patients when associated with TMZ therapy (p < 0.05).

All other prognostic clinical parameters of glioma patients and their relation with OS and PFS are shown in Table 2.

We performed Multivariate Cox regression analysis (Table 3) for various variables where both MGMT promoter methylation as well as loss of protein its expression were significantly found to be an independent prognostic factors in glioma patients with Hazard Ratio (HR) of death as 4.15 (OS) and 0.82 (PFS) for MGMT methylation (p < 0.05) and 0.73 for expression (p = 0.002). This was in addition to significant concordance with TMZ therapy and radiotherapy as depicted in Table 3.

**Table 3:** Multivariate (Cox Regression Model) survival analysis of different variables and its association Glioma patients.

Variable	Cox Regression-Overall Survival			Cox Regression-PFS Survival				
	HR	95.0% CI		P value	HR	95.0% CI		P value
		Lower	Upper			Lower	Upper	
Age years	0.74	0.37	1.48	0.40	0.35	0.41	2.00	0.80
Surviving	0.40	0.08	1.97	0.26	0.90	0.09	2.44	0.38
Grade	0.80	0.42	1.50	0.49	0.48	0.39	1.72	0.61
MGMT status	4.15	1.59	10.81	0.003	0.82	3.21	37.96	0.000
Chemotherapy	25.13	6.23	101.22	0.00	11.04	5.26	128.5	0.00
Survival	2.24	0.45	11.16	0.32	25.98	0.45	13.42	0.29
Expression	0.73	0.30	1.799	0.50	2.48	0.33	2.22	0.75
Radiotherapy	3.89	1.32	11.51	0.01	0.86	1.34	14.79	0.01
Gender	2.14	0.91	5.03	0.08	4.45	0.55	4.12	0.42

HR: Hazard ratio.

Again by multivariate Cox regression model (Table 4), correlation was evaluated between the status of TMZ therapy and MGMT parameters (methylation and its expression), with other characteristics taken into consideration including age at first contact, and gender. TMZ therapy had a significant impact on glioma patients with MGMT promoter methylation with a HR of 0.76 (0.21–2.899; p = 0.029) as compared to 3.78 HR (95%; 1.71–6.8); p = 0.70 for those who did not receive therapy achieving an independent prognostic significance as indicated in Table 4. Similarly, lack of MGMT protein expression showed significant concordance along with methylation pattern when correlated with TMZ therapy (HR = 2.02 (95% C.I; 2.00–5.847; p = 0.04).

**Table 4:** Multivariate analysis for determination of Survival in Glioma patient cohort treated with TMZ therapy status.

Variable	TMZ Therapy			No TMZ Therapy		
	HR	95% CI	P-value	HR	95% CI	P-value
Age	1.01	0.97–1.059	0.599	1.05	1.00–1.0	0.036
Gender	0.05	0.004–0.645	0.022	0.24	0.08–0.7	0.014
MGMT Protein Expression	2.02	2.00–5.847	0.040	4.31	2.09–7.0	0.006
MGMT Promoter Methylation	0.76	0.21–2.899	0.029	3.78	1.71–6.8	0.706

HR: Hazard ratio; TMZ: Temozolomide.

## Discussion

MGMT promoter gene methylation followed by its subsequent protein inactivation has been substantiated to modulate response of chemotherapeutic drugs like alkylating agents (Temozolomide) commonly used in malignant gliomas. A number of clinical trials authenticated that MGMT methylated sequence serves as a strong prognostic factor for longer survival and progression free disease^25–29^. The current study investigated the concordance between MGMT gene methylation status and its protein expression to determine their correlation with TMZ therapy in a series of glioma patients of varied histologies. Most of the studies previously done have taken selected histological types of malignant gliomas, but we focused almost on all the types of glioma where TMZ therapy is indicated as part of the chemotherapy (included GBM, Astrocytoma, Oligidendrioglioma etc.)

Our study found a great deal of concordance between the MGMT gene methylation and its protein expression (82%). Normally promoter methylation of MGMT silences gene activation resulting in loss of its protein synthesis and this assessment can be authenticated through protein expression to assess MGMT gene status. The MGMT gene produces DNA repair protein that is confirmed to confer resistance against cellular toxicity by alkylating agents^30^. Therefore, intact MGMT protein in relation to TMZ therapy provides therapeutic detrimental protective impact and conversely is confirmed not to be present when MGMT protein is lost owing to epigenetic silencing of its promoter as seen in various tumors in particular glioma, thus rendering cells more sensitive to alkylating agents. The sensitivity of MGMT promoter methylation analysis was improved in this study by selecting quality DNA, extracted from fresh frozen tissues coupled with the assessment of effective CpG sites. Furthermore, to avoid the statistical bias for predictive value of MGMT gene parameters owing to tumor heterogeneity, a re-sampling analysis was done which substantiated for reproducibility of the results.

The frequency of MGMT methylation status has shown discrepancies in glioma across the globe. In our study we found 60.3% cases with MGMT methylated sequences as compared to 39.7% unmethylated DNA wherein GBM accounted for 53.1% with methylated DNA followed by Astrocytoma 64.2% and Oligidendrioglioma 85.7%. This pattern is in accordance with other studies where MGMT promoter is reported methylated in 30–60% of GBM^31^ and in 30–90% of low-grade gliomas including Astrocytoma and Oligidendrioglioma^13,17,21^. It shows that in a large subgroup of patients, MGMT gene with methylated sequences unambiguously serves as the genetic fingerprint with highest clinical impact on outcome of the glioma patients when treated with Temozolomide and radiotherapy. An important finding from this study is the highly significant concordance between the status of MGMT methylation and its *in situ* protein expression by IHC (63% MGMT hypermethylation v/s 60% loss of MGMT protein; p = 0.000). It is evident that methylation of MGMT gene in the promoter region like many other genes is associated with the silencing of the gene and correlates with loss of transcripti^14,15,32–34^. The concordant correlation between MGMT methylation and subsequent loss of its protein has been substantiated in many reports on tumors which augment the reason for the loss of MGMT activity^35–38^. Many previous reports agree with our study where a significant correlation of MGMT parameters was found^39,40^ but still some studies are in contrast to this observation^33,34,39–41^. The concordance found in our study between MGMT methylation and loss of expression as also reported earlier^13^ provides a direct link of these two events and suggests a valuable role for aberrant promoter hypermethylation of MGMT in primary human cancer^13^. The least variability for MGMT detection by these two methods helped us to determine the clinical outcome of the same series of patients treated with TMZ therapy and radiotherapy. There is a rarity of clinically applicable molecular biomarkers to predict the treatment outcome due to complex nature of cancer^42^ including malignant Glioma. MGMT gene has been successfully substantiated in many investigations to be applied as a therapeutic target when downregulated to increase the chemosensitivity of malignant gliomas to alkylating drugs (TMZ). Therefore, evaluation of specific and capable biomarkers intrinsic to cancer hallmarks, like the prognostic role determined by MGMT gene in our study, becomes mandatory subject to its ability to aid in cancer prediction.

A milestone study by Hegi *et al*. (2005)^17^ shows some semblance with some other studies, but some discrepancies do exist in other reports which lacked association between MGMT methylation and clinical outcome of TMZ therapy in high grade malignant gliomas^17,43–45^. The reason would be either sample size or the method of MGMT parameter evaluation. In our series, glioma patients with methylated MGMT sequences were seen as strong prognostic factor in terms of better OS and PFS of 40 and 23.9 months versus 6.7 and 3.3 months in unmethylated MGMT respectively (p = 0.000) which is in accordance with previous reports^46,47^. Similarly lack of MGMT protein also correlated with better OS and PFS of 36.2 and 22.6 months as against 18.2 and 8.9 months respectively in patients with intact protein (p = 0.001). Both MGMT parameters proved to be significant prognostic factors in our series of glioma cases across different histological types of glioma. A better OS and PFS was noted for patients treated with TMZ therapy as compared TMZ untreated and same was true for the use of radiotherapy (p = 0.000). It is evident from the results as supported by other studies that MGMT methylation with concordant loss of MGMT protein play an important role in modulation of chemotherapeutic response (TMZ therapy). The recent revelations of epigenetic involvement in cancer development and treatment owing its role in regulation of plethora of genes make a ground for the development of cancer hallmarks. Biomarkers have been important in our knowledge of gliomagenesis and though meager, are robustly being used for stratification of different glioma hiostologies, diagnosis and treatment for predictive relevance. In accordance with potential impact of MGMT gene for predictive value, Gao S *et al*. (2016) recently developed efficient cancer hallmark–based gene expression signature sets that could specifically stratify a subset of colorectal cancer patients with stage II disease to benefit long survival from adjuvant chemotherapy and thus predict good prognosis^42^.

Survival analysis of our studied patients followed prospectively showed slightly longer survival in GBM (13.8 months) than other retrospective study which reported a shorter OS for GBM patients (around 10 months)^4^ but is in agreement with our investigations (14.6 months and 12.1 months). Although the robustness of MGMT gene as a predictive marker was evident in our series of glioma patients but still tumor heterogeneity casted doubt on its efficiency as reported in an earlier study also^48^. To address this, we conducted a comprehensive re-sampling analysis to analyze MGMT methylation and expression to correlate with patient survival. The data set obtained although did show some deviation from the initial analysis but overall did not influence the significant impact of MGMT signature on treatment outcome and survival. This aided us in further fitting the data for the refinement of predictive power of MGMT gene parameters in Glioma patients and their treatment outcome. In this context another study investigated 26 S proteasome genes as a prospective prognostic signature by expression levels and the survival analysis depicted this marker could discriminate between the breast tumor patients with long and short survival^49^. Prognostic biomarkers if interpreted properly to address data over fitting can act as potential tools for prediction of survival subsequent to diagnosis of cancer and aid in its management for best outcome.

In multivariate analysis, we notably detected a significant impact of TMZ therapy on the patients who presented with methylated MGMT and lack of its protein expression on OS and PFS than those who did not receive this treatment. The trend indicated that TMZ therapy in our patients indicates a strong favorable outcome as demonstrated by earlier studies where a high frequency of MGMT promoter methylation was observed in longer OS of GBM put on repetitive TMZ therapy^4,47^. Though in sync with our report that MGMT methylation favors longer survival in glioma patients treated with TMZ therapy^4,47,50–52^ but notably survival was longer in our series of patients. We found patients with lack of MGMT protein who were put on TMZ therapy had better survival than those without (37.3 v/s 9.5 months; p = 0.002) and similarly MGMT methylated subgroup on TMZ therapy had longer OS against those not treated (44.0 v/s 11.2 months; p = 0.002, Fig. 4). This analysis confirms for the first time a strong significant concordance between the MGMT parameters (methylation and loss of protein) for better survival in patients treated with TMZ therapy. In agreement to this, a previous report has also authenticated that MGMT protein status by IHC associated strongly with MGMT methylation in GBM cases^53^. Some reports predicted MGMT protein expression to benefit with TMZ therapy for survival in malignant glioma^54–56^ giving a notion that only the protein levels are associated with MGMT activity. Further in partial agreement with our study Nagane *et al*. (2007) reported that MGMT protein expression by IHC rather than its methylation pattern depicted as an independent favorable prognostic factor for OS of patients treated with TMZ^57^. In yet another study by Nifterik *et al*. (2010) from GBM cell lines including other tumors reported MGMT loss of protein as a favorably better prognostic factor than its methylation status with respect to TMZ therapy response^58^. Though cancer cell lines prove effective models for the *in vivo* investigation, but results need to be authenticated in patient sample series, and so far immunohistochemical evaluation of the MGMT protein level in tumor samples have come up with inconclusive findings when correlated with patient outcome. It is a fact that MGMT expression analysis by IHC in certain reports showed lack of association with chemotherapeutic response and OS of patient^37^, but it can be possibly associated with some methodical errors owing to different cell morphology and mixed infiltrative staining cells in glioma cases etc^4^. The discrepancy is evident as we go through the relevant studies where some have shown MGMT methylation and others its protein loss as prognostic factor with respect to clinical outcome. Interestingly, this study in comparison to other reports investigated the different groups of glioma cases and found synchronized pattern of both MGMT parameters with a better response to TMZ therapy and favorable long term survival. Epigenetic alterations like MGMT lesions and its role in drug therapy provides a platform to conceptualize mechanistic models of their predictive values to reach a census to affiliate for cancer hallmark. To this end we have done re-sampling analysis through a valid statistical tool for all the variables of the study to derive estimates of bias, standard errors and confidence intervals for proportions in case and controls to check the stability of results. We found the reproducible predictive power of MGMT gene parameters to quantify its ability as cancer hallmark for its molecular and expression phenotypes of malignant glioma cells. Previously, an operational signature of survival networks has been developed to predict drug targets for specific clones within a tumor^59^. These valuable analysis tools help to demonstrate the modality to use the hallmark network to predict efficacy of chemotherapeutic drugs.

Further, on methodological aspects more refined MGMT protein detection by IHC or any other method or a tool may help to improve the results in protein validation for effective interpretation for TMZ therapy response in glioma. In recent findings, *MGMT* gene promoter methylation also predicts patients whose tendency to have a greater probability of a radiographic pseudoprogression when put on Temozolomide^28,18^ but gradually improves with continued therapy.

Since MGMT epigenetic analysis and protein expression is unambiguously the best genetic signature with respect to clinical outcome of glioma patients than evaluations of its mRNA^45^, a more reliable and reproducible methods to detect it may be developed to further augment the management of the disease with respect to chemotherapy by alkylating agents.

We summarize that MGMT parameters, methylation and loss of its protein were found as strong prognostic predictive factors while using TMZ therapy showing beneficial survival and progression free survival. We conclude both parameters of MGMT should be confirmed to benefit the glioma patients while deciding for chemotherapy with Temozolomide to manage the disease efficiently.

## Methods

### Patients

The present study was conducted in Department of Advanced Centre for Human Genetics, Sher-i-Kashmir Institute of Medical Sciences (SKIMS), Srinagar (J&K, North India) between 2013 and 2017. The patients with malignant glioma were included after written informed consent. The surgically resected tumor tissue samples taken through stereotactic/open biopsy of brain tumors, were collected directly into sterile vials containing chilled PBS (pH = 7.2) and frozen at −70 °C for molecular investigations and samples for IHC were collected in formalin.

All glioma patients seen consecutively in Medical Oncology and Neurosurgery departments of SKIMS were included in the study and the sample size was calculated as per the hospital records which showed a power of the study >75. Glioma patients were assessed for detailed history, physical and systemic examination.

All the patients were evaluated by radiological examinations by X-ray chest, CECT brain and contrast enhanced MRI brain. The patients were subjected to gross total resection, subtotal resection or biopsy depending on the patient’s status and tumor location. All tumor samples were histologically confirmed as malignant gliomas and classified by two expert Neuropatholigists at the Department of Pathology in SKIMS according to the WHO criteria of CNS using formalin-fixed, paraffin-embedded specimens.

Once the pathology was confirmed, all the patients were put on Temozolomide^16^ and radiotherapy was delivered as per the hospital protocol. Patients were treated with concurrent chemo-radiotherapy that included Temozolomide with megavoltage radiation therapy. Radiotherapy was delivered as 60 Grays in 30 fractions at 2 Gray per fraction, five days a week for a period of six weeks.

The gross tumor volume was determined by pre and post-operative MRI imaging using enhanced T1 and FLAIR/T2. The Gross Tumor Volume (GTV) was expanded by 2 to 3 cm to generate Clinical Target Volume (CTV), to account for sub-diagnostic tumor infiltration. Radiation fields were reduced after 46 Grays to prescribe boost radiation to gross disease.

Patients received oral Temozolomide 100 mg/m2 per day for the duration of radiotherapy. Three to four weeks after radiotherapy, patients were given TMZ 150 to 200 mg/m2 on days 1 through 5 in 28-day cycles for a maximum of 12 cycles in patients with complete response (CR) and up to progression in patients with partial response (PR) or stable disease. Hematologic tests were done at baseline and on days 21 and 28 of each cycle. All patients received oral premedication during treatment. Patients were evaluated for follow up MRI/CECT head at 1st week and every three months thereafter. Other biochemical parameters like electrolytes, renal and liver functions were examined at baseline and on day 21 of each cycle. Patients were followed-up by imaging like magnetic resonance image every 3 months^22^.

The response to treatment TMZ used for malignant glioma as chemotherapeutic agent was analyzed in the backdrop of MGMT status corresponding with the treatment in terms of overall survival (OS) and progression free survival (PFS). Survival time of the patients was deduced or reckoned from either the date of diagnosis or the start of treatment for the disease up to the time they continue to survive. Survival time was also determined from the date of first surgery up to death or date of last contact if lost to follow up evaluation. Follow up ranged from 6 months to a maximum of around 36 months.

Histopathologically confirmed different types of malignant glioma tissues and corresponding normal tissues were used for MS-PCR and paraffin embedded tissue (FFPE) samples of the corresponding patients were subsequently used for IHC.

### DNA extraction of glioma tumor tissues

Fresh frozen malignant glioma tissue samples were subjected to DNA extraction using standard phenol/chloroform methods. The purity and concentration of DNA was estimated at the absorbance of 260 and 280 nm and checked on 1% nuseive agarose gel.

### Bisulphite DNA Modification and Methylation-Specific Polymerase Chain Reaction (MS-PCR)

1.5 to 2 µg of DNA was treated with chemical bisulfite modification (EZ DNA Methylation Kit,Zymo Research Corporation, USA) to convert unmethylated, but not the methylated, cytosines to uracil. Subsequently PCR was employed using 2 µl of modified DNA by primers specific for either methylated or the modified unmethylated DNA (16) containing 20 pmol of primers (Zymo Research Corporation, USA), 1.25 mM MgCl_2_, 10x Reaction buffer, 1 U *Taq DNA Polymerase* and 200 mMdNTPs (Invitrogen, Karlsruhe, Germany). PCR was performed with thermal conditions as: 95 °C for 5 minutes, 30 cycles of 94 °C for 35 seconds, 52 °C for 45 seconds and 72 °C for 35 with a final extension of 72 °C for 7 minutes. The PCR reaction was carried out in two separate tubes specific for methylated and unmethylated sequences. The PCR products were run on a 2% agarose gel yielding a band of 81 bp for methylated and 93 bp for an unmethylated product. The agarose gel bands were documented using the Flourchem HD2 (Cell Bioscience, USA).

### Immunohistochemistry (IHC) for MGMT protein

FFPE tumor blocks were analyzed by IHC using 4 µm sections. Antigen was retrieved in 10 mM citrate buffer (pH 6.0) at 95 °C to 122 °C for 15 minutes in EZ Retriever system (BioCare Medical, Walnut Creek, CA). Slides (sections) were deparaffinized at 60 °C followed by xylene changes. Endogenous peroxidase activity was blocked with 0.3% H_2_O_2_ in methanol for 20–30 min. Slides were incubated at 4 °C overnight with a mouse antibody against human MGMT (primary antibody) (Santacruz, Biotechnology, California, USA) used at final dilution of 1:250 to 1:500. Diaminobenzidine was used to catch the signal followed by a nuclear stain (hematoxylin:blue).

Assessment and scoring of MGMT expression in tumor sections was performed by two independent pathologists who were blinded to methylation pattern and other parameters with scoring method based on percent of cell nuclei that were positive: 0 (no staining), 1 (10–25%), 2 (26–50%), or 3 ( > 50%).

Staining for MGMT protein was considered positive when uniform MGMT staining was displayed in the nuclei of cells. Staining that was restricted to the cytoplasm and granular nuclear reactivity was considered negative.

For statistical analysis, scores of 0 were defined as the absence of protein expression, and scores of 1 to 3 were considered as positive for protein expression.

### Statistical Analysis

The patients were followed up to determine the Overall survival (OS) from the date of the diagnosis while as progression-free survival (PFS) was deduced at the time patient developed progression as a new lesion or when there appeared some neurologic disturbances like impaired memory or locomotor dysfunction. Different tests for homogeneity of proportions including Chi square and Kaplan Meier analysis to evaluate survival outcome probabilities were used to determine significance of the distribution patterns with respect to different clinico-analytical parameters. Statistical analysis was performed by using IBM Statistics SPSS software (Version-23). Statistical significance was set at the level of P < 0.05.

### Ethical approval

All procedures performed in studies involving human participants were in accordance with the ethical standards of the institutional and/or national research committee and with the 1964 Helsinki declaration and its later amendments or comparable ethical standards and ethical approval was obtained from Institutional Ethical Committee (SKIMS Study ref: Protocol 81/2013).

## Electronic supplementary material

supplementary figures and tables
