# Supplementary material for: Concordant association validates MGMT methylation and protein expression as favorable prognostic factors in glioma patients on alkylating chemotherapy (Temozolomide)
Source: Sci Rep. 2018 Apr 30;8:6704. doi: 10.1038/s41598-018-25169-2 (PMC5928198; doi:10.1038/s41598-018-25169-2)

Concordant association validates MGMT methylation and protein expression as favorable prognostic factors in glioma patients on alkylating chemotherapy (Temozolomide)

Arshad A. Pandith[1*Ψ], Iqbal Qasim[1Ψ], Wani Zahoor[1], Parveen Shah[2], Abdul R Bhat[3], Dheera Sanadhya[4], Zafar A Shah[5], Niyaz A Naikoo[6],

Ψ Authors contributed equally

[1]Advanced Centre for Human Genetics, Sher-I-Kashmir Institute of Medical Sciences (SKIMS), Srinagar, J&K-India

[2]Department of Pathology, SKIMS, Srinagar, J&K-India

[3]Department of Neurosurgery, SKIMS, Srinagar, J&K-India

[4]School of Life and Basic Sciences, Jaipur National University, Jaipur-302025, India

[5]Immunology and Molecular Medicine, SKIMS, Srinagar, J&K-India

[6] Department of Biotechnology, Higher Education Department, Cluster University, J&K-India

***Corresponding Author**

Dr. Arshad Ahmad Pandith, PhD

Advanced Centre for Human Genetics

Sher-I-Kashmir Institute of Medical Sciences, Srinagar, Kashmir, India

Email: arshaajiz@gmail.com

arshad.pandith@skims.ac.in

Tel: 01942401014: (Ext: 2414)

Supplementary Table 1: Distribution analysis of selected demographics factors in glioma cases

Demographic feature	Glioma cases n= 63	Percentage %
**Age**		
≥50	30	47.6
<50	33	53.3
**Sex**		
Male	47	74.6
Female	16	25.4
**Residence**		
Rural	25	39.7
Urban	38	60.3
**Tumor Type**		
Glioblastoma	32	53.9
Astrocytoma	14	22.2
Oligidendrioglioma	14	22.2
Others	03	4.7
**Grade**		
I/II (Low)	6	9.5
III	27	42.9
IV (High)	29	46.0
**Chemotherapy**		
Temozolomide Given	45	71.4
Temozolomide Not Given	18	28.6
**Radiotherapy**		
Given	42	66.7
Not Given	21	33.3
Chemotherapy + Radiotherapy	37	58.8
Chemotherapy Alone	05	7.9
Radiotherapy Alone	04	6.4
No therapy	17	26.9
**Vital Status**		
Dead	36	57.1
Alive	27	42.9

Supplementary Table 2: MGMT promoter methylation and protein expression status with respect to different parameters of Glioma patients

Parameter	MGMT Promoter Methylation		P-value	MGMT Protein Expression		P-value
	Unmethylated n=22	Methylated n=38		Negative n = 36	Positive n=24	
**Age**						
≥50	12	18	0.9	17	13	0.5
<50	13	20		19	11	
**Sex**						
Male	16	29	0.7	26	18	0.5
Female	06	09		10	04	
**Tumor Type**						
Glioblastoma	15	17	0.1	19	13	0.7
Astrocytoma	05	09		09	05	
Oligidendrioglioma	02	12		08	06	
**Grade**						
Low	03	03	0.5	04	02	0.9
III	08	19		17	10	
IV (High)	11	16		16	13	
**Chemotherapy**						
Temozolomide Given	09	30	0.003	34	09	0.01
Temozolomide Not Given	13	08		02	15	
**Radiotherapy**						
Given	09	33	0.000	31	11	0.02
Not Given	13	05		05	13	
Chemo + Radio	07	30	0.000	28	09	0.000
Chemotherapy Alone	0 2	03		04	01	
Radiotherapy Alone	02	01		02	02	
No therapy	13	04		02	12	

Supplementary table 3: Correlation of MGMT methylation and its protein expression in the backdrop of TMZ therapy status

Treatment	MGMT Methylated	MGMT Unmethylated	P value	MGMT expression	MGMT No Expression	P value
**Overall status**	30	09	0.007	09	34	.01
TMZ +	08	13		15	02	
TMZ-						
**Glioblastoma**						
TMZ +	17	01	0.000	02	18	0.000
TMZ-	03	11		10	02	
**Astrocytoma**						
TMZ +	7	2	0.08	01	08	0.09
TMZ-	2	3		03	02	
**Oligidendrioglioma**						
TMZ +	7	3	0.9	03	07	0.6
TMZ-	2	2		01	02	
**Low grade**						
TMZ +	2	2	0.9	01	04	0.9
TMZ-	1	1		01	01	
**High grade**						
TMZ +	30	06	.0001	05	31	0.000
TMZ-	05	13		12	04	
**<u>Dead</u>**						
TMZ +	13	2	0.000	02	15	0.000
TMZ-	03	12		13	03	
**<u>Alive</u>**						
TMZ +	20	2	0.02	03	20	0.6
TMZ-	02	2		02	01	
**Female**						
TMZ +	10	1	0.02	02	10	0.5
TMZ-	1	3		02	02	
**Male**						
TMZ +	21	04	0.00	05	23	0.00
TMZ-	5	10		11	05	

Supplementary fig1: Association of OS and PFS with respect to Temozolomide therapy

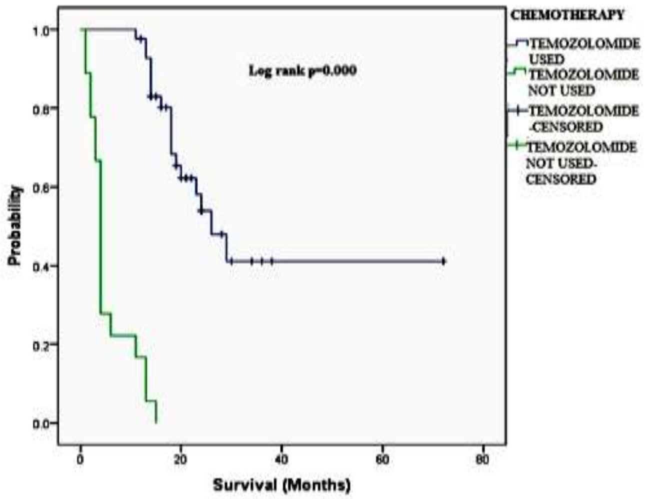

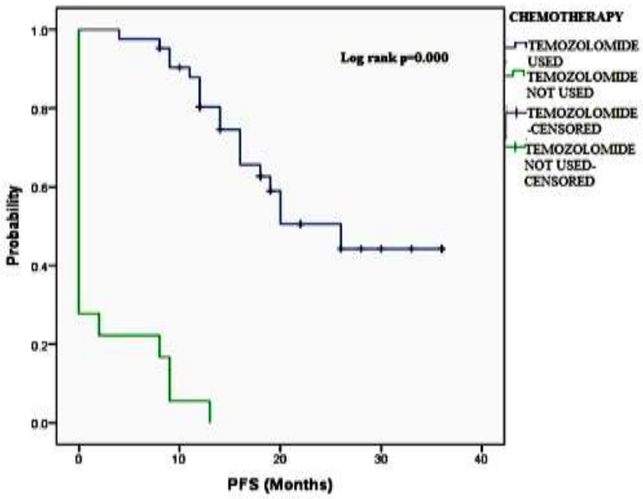

Supplement: Supplementary file 1 — supplementary figures and tables [file 41598_2018_25169_MOESM1_ESM.pdf]
